# Effects of Slaughter Methods on the Quality and Refrigerated Shelf Life of Biofloc-Cultured White Shrimp (*Penaeus vannamei*)

**DOI:** 10.3390/foods15101695

**Published:** 2026-05-12

**Authors:** S. Ferrando-Juan, A. Honrado, A. Tomás-Vidal, S. Martínez-Llorens, M. Rodilla, M. Jover-Cerdá, J. A. Beltrán Gracia, D. S. Peñaranda, J. Calanche

**Affiliations:** 1 Aquaculture and Biodiversity Research Group, Institute of Science and Animal Technology (ICTA), Universitat Politècnica de València, Camí de Vera, s/n, 46022 València, Spain; sferjua@doctor.upv.es (S.F.-J.); atomasv@dca.upv.es (A.T.-V.); silmarll@dca.upv.es (S.M.-L.); mjover@dca.upv.es (M.J.-C.); dasncpea@upv.es (D.S.P.); 2 Animal Production and Food Science Departament, Faculty of Veterinary Medicine, AgriFood Institute of Aragón (IA2) Miguel Servet, 177, 50013 Zaragoza, Spain; adrihonfri@unizar.es (A.H.); jbeltran@unizar.es (J.A.B.G.); 3 Research Institute for Integrated Management of Coastal Areas, Universitat Politècnica de València, Carretera de Nazaret-Oliva, s/n, Grao de Gandía, 46730 València, Spain; mrodilla@hma.upv.es

**Keywords:** biofloc technology, slaughter technique, spoilage, melanosis, sensory, quality, shelf-life, white shrimp

## Abstract

Ethical slaughter practices for crustaceans remain poorly standardized, and their effects on product quality and consumer perception are insufficiently understood. This study evaluated four ice-slurry-based slaughter methods in *Penaeus vannamei* reared under intensive biofloc technology (BFT) and their impact on refrigerated shelf life. While BFT enhances farming sustainability, it may an increase in microbial load, potentially shaping post-mortem spoilage. Shrimp were subjected to cold thermal shock in seawater ice slurry (C-TS); a 5 min immersion in chilled seawater with 50 ppm sodium hypochlorite, followed by TS (5BTS); 3–4 h of intestinal clarification, followed by 1 min immersion in chilled seawater with 50 ppm sodium hypochlorite and subsequent TS (C1BTS); and TS, followed by UV-C exposure (TS-UV). Over 12 days at <4 °C refrigeration, culture-based microbiology (mesophilic bacteria, enterobacteria, psychrotrophs, pseudomonas, and specific spoilage organisms), total volatile nitrogen compounds (TVB-N), melanosis, and qualitative descriptive analysis were performed. Storage time showed typical spoilage patterns driven by psychrotrophic bacteria, TVB-N, and melanosis. Slaughter method influenced quality: C1BTS limited psychrotroph proliferation but accelerated melanosis, and TS-UV yielded the poorest performance. Notably, 5BTS delayed melanosis (~3 days), maintained sensory quality, and avoided excessive microbial growth, making it the most effective method. These findings provide practical guidance for additive-free shrimp processing and identify psychrotrophs as key spoilage indicators in BFT systems.

## 1. Introduction

Aquatic products, particularly shrimp, are preferred by a great number of populations due to their nutritional contribution: high protein content, low fat, vitamin source, and easy digestion [1]. The Pacific whiteleg shrimp (*Penaeus vannamei*, Boone 1931) is the second most important aquaculture animal species worldwide, with a production of 7.4 million tons in 2023 [2]. Moreover, it is the species with the highest economic value within the sector, reaching a production value of €35.398 billion, representing an increase of 13.3% compared to 2022 [3]. As consumer demand and market growth continue to rise, the shrimp industry faces increasing pressure to ensure sustainable production practices, maintain product quality, and adhere to ethical standards regarding animal welfare throughout farming and processing.

For decades, shrimp and other crustaceans were assumed incapable of experiencing pain [4], and consequently, standardized slaughter methods have not been established. However, growing societal concerns regarding animal welfare, together with increasing scientific evidence supporting the potential sentience of decapod crustaceans, have driven a shift in this perception. This is reflected in recent regulatory developments such as the Animal Welfare (Sentience) Act 2022 [5], which recognizes decapod crustaceans as sentient animals capable of experiencing pain and distress. In parallel, consumer expectations for freshness and product quality have prompted interest in more ethical slaughter practices based on the principle that animals should be killed rapidly and with minimal pain and suffering (European Union Council regulation 1029/2009 and no 853/2004) [6,7]. Slaughter techniques are diverse because crustacean species vary greatly in their physiological and anatomical characteristics. Several methods have been explored in crustaceans, including freezing, superchilling with liquid nitrogen, salt baths (MgCl_2_), CO_2_ exposure, electrical stunning, chilling in air or ice slurry, and boiling. Each presents specific advantages and limitations in terms of efficiency, practicality, and animal welfare [8]. Among these, immersion in ice slurry has emerged as one of the most recommended practices, as it induces sedation with a rapid decline in cardiac activity in *P. vannamei* [9], leading to death within minutes. Importantly, this method must be carried out in seawater-based ice slurry to avoid osmotic shock caused by freshwater ice melt [10].

Shrimp are highly perishable products, and their quality and shelf-life depend on both internal and external factors, such as the chemical composition of the flesh, physiological condition, and the methods of culture and handling (including slaughter, storage, and transport) [11]. Fresh shrimp typically exhibit a bright natural color, pleasant odor, and firm texture [12]. However, due to their high water content, they undergo rapid post-mortem changes in texture, color, and flavor, driven by enzymatic activity, protein degradation, lipid oxidation, and microbial metabolism [13,14]. Consumer acceptance and, consequently, shrimp market value are mainly determined by visual appearance. One of the main contributors to sensory deterioration is melanosis, or black-spot formation, an enzymatic browning process that causes post-mortem darkening of the shrimp [15]. Although melanosis becomes noticeable only a few hours after harvest and does not pose a safety concern, it represents a major quality defect [16].

Quality changes in shrimp can be assessed using different approaches, including microbial, chemical, and sensory analyses [17]. These analyses typically involve measuring bacteria associated with process and handling, such as *Staphylococcus aureus*, *Escherichia coli*, *Salmonella* sp., *Listeria monocytogenes*, or *Pseudomonas* sp. [18]; determining volatile compounds like trimethylamine and total volatile basic nitrogen [19,20]; and evaluating sensory attributes such as appearance, odor, texture, and taste, either by trained panelists or through consumer acceptance tests, to assess product freshness and spoilage [21]. Traditionally, research has focused on post-harvest handling, storage, and processing practices. For instance, some studies have assessed the quality and consumer acceptability of fresh *P. vannamei* stored at ambient temperature during the first 20 h post-mortem to promote local consumption near production areas [20]. Others have examined how different refrigeration temperatures (0–4 °C) affect shrimp quality during storage [22,23]. Additionally, several works have explored treatments applied during rigor mortis to extend product shelf-life [24,25]. However, despite the critical role of slaughter as the first step in the post-harvest chain, little research has addressed how slaughter methods may affect shrimp freshness, quality traits, and sensory acceptance. To date, only a limited number of studies have considered related aspects. For instance, the effects of slaughter by ice-water immersion and individual beheading in *P. vannamei* have been examined using infrared spectroscopy [26], while the quality and shelf life of fresh shrimp preserved on ice, without prior treatment, have also been evaluated [27]. Nevertheless, neither of these studies assessed the impact of slaughtering methods on organoleptic properties characteristics and melanosis development on sensory evaluation.

Additionally, culture conditions themselves may also influence product quality. Currently, more than half of the shrimp consumed worldwide is produced through aquaculture, mainly in ponds, using either conventional farming systems with daily water exchange or biofloc technology (BFT), a more sustainable alternative. BFT has been widely adopted in shrimp farming, as it enhances productivity and growth performance, improves water quality within a closed system, and provides a supplemental feed source for shrimp [28,29]. BFT systems are composed of bacteria, microalgae, fungi, protozoa, and invertebrates that recycle nitrogenous waste into microbial biomass [30]. The aggregation of these microorganisms with organic particles leads to the formation of bioflocs [31]. This complex microbial community modulates associated gut microbiota in shrimp and stimulates their immune system by enhancing nonspecific immune activity [32]. However, such systems may also raise concerns about the potential accumulation of pathogenic microorganisms, while also influencing the baseline microbial load in shrimp, factors that could have implications for post-harvest quality and freshness stability [33].

Therefore, given the lack of information, the aim of the present study was to evaluate the effect of different ice slurry slaughter methodologies on the quality and shelf life of fresh *P. vannamei* cultivated in a BFT system. Specifically, microbiological and physicochemical parameters were assessed, alongside consumer acceptance, through sensory evaluation and melanosis development.

## 2. Materials and Methods

### 2.1. Ethics Approval

The present study adhered to the necessary considerations for animal experimentation, as outlined in Annex IV of RD 53/2013. However, shrimp are not included in the list of species under Council Directive 2010/63/EU and therefore do not require approval from the Ethics Committee for their handling and experimentation. The trial was conducted in collaboration between the Aquaculture Laboratory of the Universitat Politècnica de València (UPV), which holds accreditation number ES 46 250 0001091, where shrimp were reared and slaughtered, and the Meat and Fish Laboratory and Pilot Plant of the Faculty of Veterinary Medicine at the University of Zaragoza (UNIZAR), where the shelf-life assessment, including microbiological tests, physicochemical analysis, and a Quantitative Descriptive Analysis (QDA) were performed. Sensory analysis was conducted in accordance with the ethical principles of the Declaration of Helsinki. Participants were fully informed about the purpose of this study and provided written informed consent prior to participation, with the option to withdraw at any time. This study involved minimal risk, as it consisted of standard food consumption under controlled conditions. No sensitive personal data were collected, and all data were anonymized and handled in accordance with the General Data Protection Regulation (EU) 2016/679 [34], and the Spanish Organic Law on Data Protection and Guarantee of Digital Rights (LOPDGDD 3/2018) [35]. This is stated in the manuscript.

### 2.2. Experimental Design

#### 2.2.1. Animals and Facilities

Shrimp (*P. vannamei*) were reared under super-intensive conditions at a density of 350 kg/m^3^ in a BFT system, consisting of an indoor, isolated, and independent 3 m^3^ tank. The physicochemical parameters of the biofloc were as follows: temperature of 28 °C, salinity at 21 g/L, alkalinity ranging from 150 to 300 mg/L, pH between 7.5 and 8.5, dissolved oxygen (DO) level greater than 5 mg/L, and natural photoperiod [36]. To ensure uniformity across the experimental units, the seawater was chlorinated with 5% sodium hypochlorite and maintained under aeration until residual chlorine levels reached 0 mg/L. Afterward, an initial inoculum of 5 mg/L of biofloc was added to promote system maturation [37]. No water renewal was carried out during the shrimp culture, and only fresh water was added to compensate for volume loss due to evaporation. Animals were fed three times a day with commercial feed (Le Gouessant, Lamballe-Armor, France) until they reached an average weight of 18 g.

During the rearing period, shrimp were subjected to routine weekly sampling, where 100 individuals were randomly collected from the tank and used to estimate average body weight and adjust feeding rates. This represented the main handling procedure applied prior to harvest. No abnormal physicochemical conditions were recorded throughout the culture period, and water quality parameters remained within optimal ranges, minimizing the likelihood of chronic stress. Additional routine handling included in situ measurement of water quality parameters using probes, which did not involve significant disturbance due to the tank size and system design.

At the end of the rearing period, the experiment was conducted as a single batch, and shrimp were randomly collected and assigned to the different slaughtering treatments to ensure comparable initial conditions. Animals were immediately processed. The time interval between harvesting and slaughter was kept to a minimum, and animals were maintained under aerated conditions during transfer to minimize stress. To minimize confounding factors, all groups were handled under the same conditions, and measurements were performed in a random order.

#### 2.2.2. Slaughter Techniques

Four slaughter methodologies based on thermal shock (TS) in ice slurry were evaluated. The ice slurry consisted of chilled seawater at 20 g/L salinity, equivalent to rearing conditions, and ice packs for maintaining temperature without diluting salinity to prevent osmotic shock. In all treatments, TS was applied as the primary method of euthanasia. Animals were divided into four groups of 50 animals each using a random numbering system.

The first approach, thermal shock alone (C-TS), used as the control, consisted of submerging shrimp in ice slurry (−2 °C) until death, confirmed by the absence of gill movement and complete body paralysis, typically within 3–4 min. In the bleach + thermal shock treatment (5BTS), shrimp were immersed for 5 min in chilled seawater (0 ± 2.5 °C) containing 50 ppm sodium hypochlorite before TS. In the clarification + bleach + thermal shock treatment (C1BTS), shrimp were first subjected to intestinal clarification by fasting in clean seawater (free of biofloc) for 3–4 h at 28 °C to allow for gut evacuation, followed by a 1 min immersion in chilled seawater (0 ± 2.5 °C) with 50 ppm sodium hypochlorite and subsequent TS. Sodium hypochlorite was applied as a short-time pre-slaughter decontamination step to reduce the high microbial load associated with biofloc systems. The selected concentration (50 ppm) was established as an intermediate level within the ranges recommended in the Codex Code of Practice for Fish and Fishery Products (CXC 52-2003) [38], which allows lower concentrations for direct contact with fishery products (~10 ppm) and higher levels for sanitation of equipment and surfaces (up to ~100 ppm). Following hypochlorite immersion, TS was performed in clean seawater ice slurry, which acted as a rinsing step to remove residual chlorine. Residual chlorine levels were monitored using a colorimetric tester, ensuring concentrations below 1 ppm prior to storage. In the thermal shock + UV treatment (TS-UV), shrimp were subjected to TS and then exposed to UV light (253.7 nm) for 2 min on each side. The treatment was performed using a Philips TUV TL Mini 8 W lamp (TUV 8 W FAM/10X25BOX, Amsterdam, The Netherlands), with a nominal UV-C output of 2.5 W (manufacturer’s specifications). The lamp was positioned at a distance of 5 cm above the samples, which were arranged in a single layer on a stainless-steel tray to ensure uniform exposure. Although irradiance at the sample surface was not directly measured, it was estimated based on manufacturer data (25 µW/cm^2^ at 1 m), resulting in an approximate range of 4–10 mW/cm^2^ at 5 cm. This estimation should be interpreted with caution, as the inverse square law provides only an approximation for tubular UV sources at short distances. After slaughter, shrimp were packed in sealed plastic bags and stored at <4 °C on fresh ice, which was replenished as needed, and melted water was removed.

### 2.3. Analytical Methods

#### 2.3.1. Microbiological Analysis

The microbiology present in the shrimp was evaluated by traditional culture-based methods. *Aeromonas* spp. and *Vibrio* spp. were evaluated qualitatively based on their presence or absence on culture plates. *Aeromonas* spp. were assessed at the genus level using Ryan agar supplemented with ampicillin, following the Ryan agar and ampicillin method [39], and *Vibrio* spp. were identified at the genus level according to ISO 21872-1:2017 [40], which does not allow species-level discrimination. The other bacterial groups were quantitatively assessed. For the initial suspension and subsequent dilutions, ISO 6887-1:2017 [41] was used. Total viable mesophilic bacteria (TVC) counts were performed at 30 °C according to the ISO 4833-1:2013 [42]. TVC were evaluated, as this parameter is commonly used in the regulation of fresh meat product quality; it serves as an indicator of the initial microbial load of the product, and controlled concentrations reflect good handling practices [43]. Although shrimp were stored under refrigerated conditions (<4 °C), TVC remains a widely accepted indicator of overall microbiological quality and was complemented by psychrotrophic counts to better reflect microbial dynamics during cold storage. The same ISO standard was followed for viable psychrotrophic bacteria (PSY), modifying the incubation temperature to 10 °C and incubation time to 4 days. The proliferation of these bacteria indicates a possible break in the cold chain and destabilization during product storage [44]. Additionally, microorganisms belonging to the *Enterobacteriaceae* (ET) family, commonly including pathogenic species such as *Escherichia coli*, *Salmonella* sp., and *Enterococcus* sp., were quantified according to ISO 21528-2:2017 [45]. Standard spoilage organisms (SSOs), indicative of product deterioration and responsible for off-odors (e.g., *Shewanella* spp.) [46], were assessed following a previously described method using Lyngby’s medium, mass homogenization inoculation, and incubation at 25 °C for 72 h [47]. Finally, *Pseudomonas* spp. (PSE) counts were determined based on ISO 13720:2010 [48], as these bacteria are associated with protein and lipid degradation and serve as indicators of spoilage [49]. All microbial counts were converted to logarithms of colony-forming units per gram (log CFU/g).

#### 2.3.2. Physicochemical Analysis

The total volatile basic nitrogen (TVB-N) content was determined following the protocol described in Commission Regulation (EC) No. 2074/2005 [50], as amended by Regulation No. 1022/2008, entitled “Determination of TVB-N concentration in fish and fishery products”, with slightly modifications. Sample amount was reduced to 1 g while keeping the ratio sample:perchloric acid at 1:10 dilution. After grinding, centrifugation, and filtration, the supernatant was mixed with 20 mL of 30% (*w*/*w*) NaOH and distilled for 3 min in a Kjeldahl unit (Velp Scientifica, mod. UDK 129, Usmate Velate, Italy). The distillate was collected in 20 mL of 3% boric acid. Titration was performed with 0.1 M HCl, using Tashiro as an indicator.

#### 2.3.3. Melanosis Assessment

**Figure A1:**
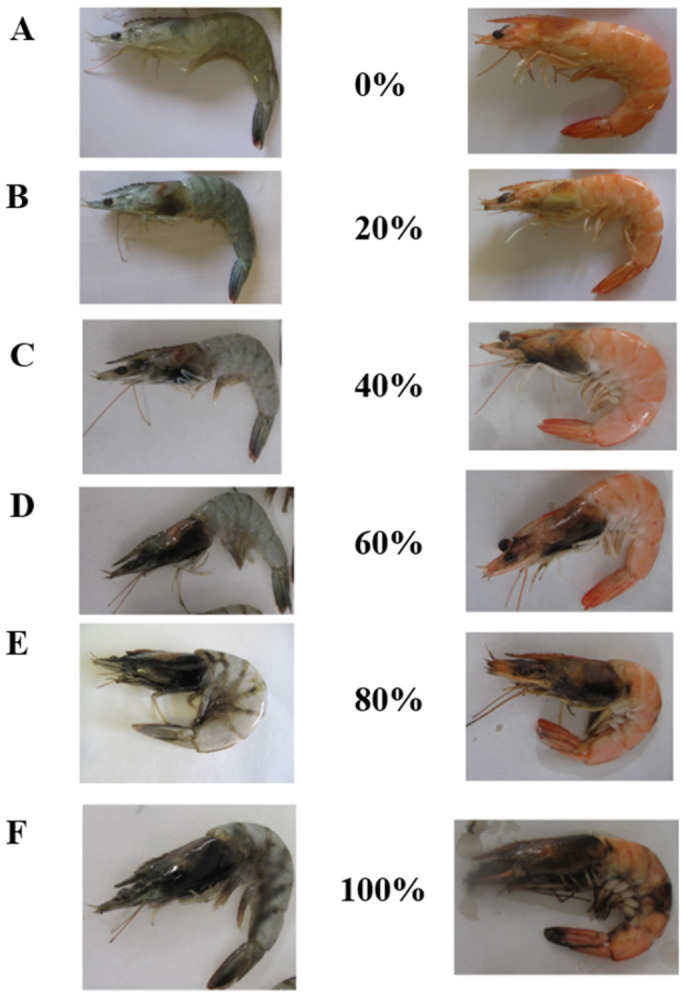
Visual melanosis assessment scale for raw and steamed *Penaeus vannamei*, ranging from 0 to 100%, corresponding to (**A**,**B**,**C**,**D**,**E**,**F**), respectively [51].

The degree of melanosis was evaluated using a visual scoring scale developed specifically for *P. vannamei* [51] based on the sensory assessment approaches previously applied to the genus *Penaeus* [16]. This scoring scheme is new and applies specifically to both raw and cooked *P. vannamei* specimens (Appendix A, Figure A1). The evaluation was carried out using randomized photographs. Each sample consisted of three shrimp, and the score assigned to each sample corresponded to the average value of the three individuals. Assessors had access to the reference scale during evaluation. The panel included 20 trained judges who underwent prior training with standardized images from separate *P. vannamei* batches. The assessment was carried out immediately following the end of the experiment.

#### 2.3.4. Sensory Quality

The quantitative descriptive analysis (QDA) in shrimp was conducted in accordance with ISO 13299:2016, following previously established methodological principles [52]. The evaluation panel consisted of 12 trained assessors from the University of Zaragoza (UNIZAR) who were previously selected and trained in accordance with UNE-EN ISO 8586:2014 [53] to ensure reliability and consistency in the sensory evaluation of shrimp. The evaluation was carried out using a structured, non-numerical scale anchored at the extremes (“absent” to “intense”) for each descriptor (Supplementary Material, sensory quality evaluation score sheet for farmed shrimp). The sensory attributes were defined during a preliminary consensus session by the panel, based on product characteristics and previous experience. Sensory sessions were performed in a standardized sensory evaluation room designed according to ISO 8589:2007 [54] guidelines. Shrimp were served at room temperature, without seasoning. Panelists were provided with water and plain breadsticks for palate cleansing between samples. The analysis was conducted <1 day (≈24 h) and 7 days post-mortem.

#### 2.3.5. Shelf-Life Evaluation

To assess the progression of product quality over time, comprehensive microbiological, physicochemical, and melanosis evaluations were performed at 0, 3, 6, 9, and 12 days post-mortem. To ensure consistency, shrimp were randomly selected while maintaining a mean body weight of approximately 18 g. For microbiological and physicochemical analyses, the second abdominal segment, including the exoskeleton, was used, following the sample-to-reagent ratios specified in the corresponding analytical protocols. For melanosis and QDA assessments, shrimp were evaluated whole, both raw and cooked. Cooking was carried out by steaming for 5 min, followed by immediate cooling in an ice bath to halt the cooking process, in accordance with Codex Alimentarius guidelines (CAC/GL 31-1999) [55].

### 2.4. Statistical Analysis

Data were analyzed using XLSTAT 2024.4.0 (Addinsoft, Paris, France). All variables studied were checked for normal distribution and homogeneity of variance using Kolmogorov–Smirnoff and Levene’s test, respectively, to detect and extract outliers. The microbiological, physicochemical, and melanosis data were analyzed using ANOVA to identify statistically significant differences among the slaughtering methods and sampling points. For microbiological and physicochemical analyses, two values per treatment and sampling day were considered, each corresponding to the mean of three replicates, while nine observations per treatment and sampling day were used for sensory data. The level of significance was set at *p* < 0.05.

Additionally, a correlation matrix (Pearson) was estimated, and Principal Component Analysis (PCA) was performed to explore the relationships between the methodologies and the parameters studied. For the sensory attributes evaluated in the QDA, a “product characterization” was conducted to highlight the sensory traits of the shrimp slaughtered using the four methodologies. This analysis also identified which sensory descriptors (organoleptic properties) that significantly contributed to discriminating among and characterizing the products obtained using the different slaughtering methodologies. In this sense, the first graph obtained shows the β coefficients for the different sensory parameters. Descriptors marked with an asterisk (*) are statistically significant in describing the characteristics of each methodology. Descriptors above the *X*-axis are considered present, while those below the *X*-axis are absent. For each slaughter method, separate graphs are provided for day 1 and day 7, representing an intra-analysis for each treatment. To summarize the results about the sensory profile of shrimp, specific graphs were obtained with adjusted means per treatment, enabling an evolution comparison from day 1 to day 7. The significant parameters were determined through the corresponding ANOVA. Descriptors marked with an asterisk (*) are statistically significant in describing the characteristics of each methodology. This provides an inter analysis, allowing comparisons between the four slaughtering methodologies.

## 3. Results

### 3.1. Microbiological Evaluation

**Figure 1:**
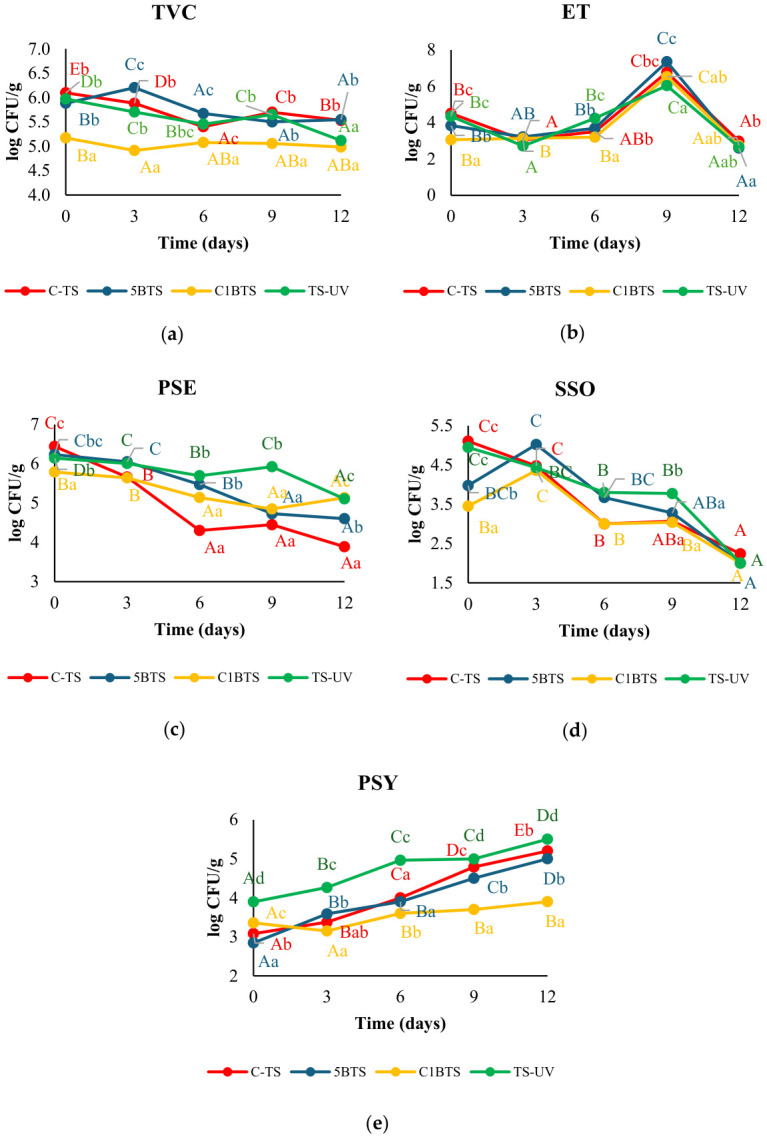
Microbial counts during 12 days of refrigerated storage in *Penaeus vannamei* subjected to different slaughtering methods: thermal shock (TS); 5 min bleach, followed by thermal shock (5BTS); clarifying plus 1 min bleach, followed by thermal shock (C1BTS); and thermal shock combined with UV treatment (TSUV). (**a**) Mesophilic bacteria (TVC). (**b**) Enterobacteria (ET). (**c**) *Pseudomonas* spp. (PSE). (**d**) Specific spoilage organisms (SSOs). (**e**) Psychrotrophic bacteria (PSY). Uppercase letters indicate significant differences among storage days, while lowercase letters indicate significant differences among treatments within each sampling day (*p* < 0.05).

*Aeromonas* spp. and *Vibrio* spp. were evaluated qualitatively based on their presence or absence on culture plates. *Aeromonas* spp. was not detected in any sample, whereas *Vibrio* spp. were consistently present in all shrimp, regardless of slaughter methodology or storage time. The presence of *Vibrio* spp. does not necessarily indicate pathogenicity, as these bacteria are commonly found as part of the natural microbiota of shrimp, particularly in biofloc systems; however, some species may act as opportunistic pathogens under conditions of microbial imbalance [56]. In contrast, the quantitatively assessed microbial groups (TVC, ET, PSE, SSO, and PSY) exhibited dynamic patterns throughout storage (Figure 1). Although several differences were statistically significant, only variations exceeding 1 log CFU/g were considered technologically relevant in terms of product quality. In relation to TVC counts, levels remained relatively stable across treatments and storage time, with an overall mean of 5.52 ± 0.06 log CFU/g. The C1BTS treatment showed consistently lower TVC values until day 3, after which counts converged with the other methodologies (Figure 1a). Regarding ET, all treatments followed a comparable temporal pattern (Figure 1b). Initial values averaged 4.01 ± 0.23 log CFU/g and remained relatively stable until day 6 post-mortem. A marked increase was observed on day 9 (6.67 ± 0.19 log CFU/g), followed by a return to baseline levels at day 12. Although C1BTS began with slightly lower ET levels, these differences were no longer evident after 3 days of storage.

PSE displayed a progressive reduction during storage across all methodologies (Figure 1c). Concentrations remained near baseline (6.15 ± 0.26 log CFU/g) until the 3-day post-mortem evaluation, after which declines occurred at different rates depending on the slaughter method: day 6 for C-TS, day 9 for C1BTS and 5BTS, and day 12 for TS-UV. Among them, the C-TS showed the most pronounced reduction, from 6.44 ± 0.11 to 3.89 ± 0.08 log CFU/g. In contrast, TS-UV maintained the highest PSE loads throughout the evaluation, until day 12, when its values approached those of C1BTS (5.12 ± 0.04 log CFU/g). For SSO, a clear comparable decreasing trend was observed (Figure 1d). Although 5BTS and C1BTS groups initially presented lower counts compared with C-TS and TS-UV, they experienced a moderate increase at day 3 and a subsequent sustained reduction. By day 12, at the end of the storage analysis, SSO concentrations had dropped by ≈3 log CFU/g across treatments (final mean: 2.07 ± 0.07 log CFU/g), with no statistically significant differences among methodologies. In contrast, PSY showed an opposite trend, increasing during storage, particularly under C-TS, 5BTS, and TS-UV treatments, where rises exceeding 2 log CFU/g were observed and considered impactful from a spoilage perspective. TS-UV registered the highest PSY counts up to day 9, after which values in C-TS and 5BTS converged. Notably, C1BTS consistently maintained the lowest PSY levels throughout the entire evaluation period.

### 3.2. Physicochemical Evaluation

**Figure 2:**
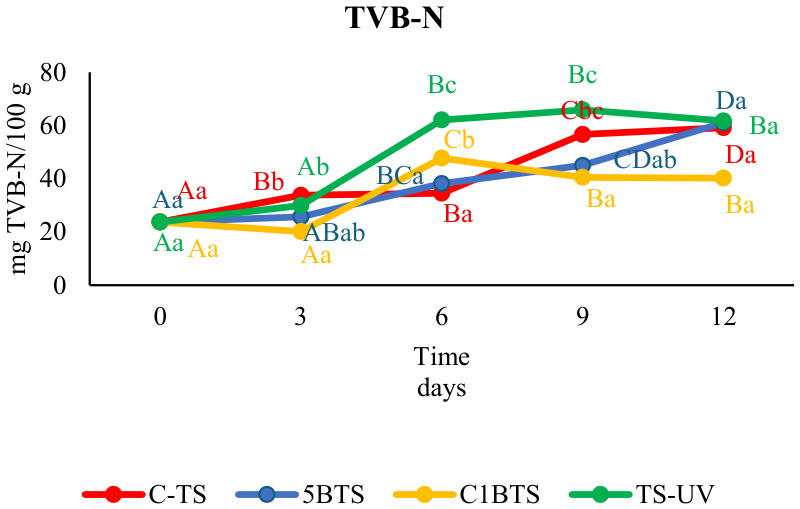
Total volatile basic nitrogen (TVB-N) values over the 12 days of post-mortem refrigerated storage for the different slaughter methodologies studied (C-TS, thermal shock; 5BTS, 5 min bleach and thermal shock; C1BTS, clarifying, 1 min bleach, and thermal shock; and TS-UV, thermal shock and UV treatment) are presented. Uppercase letters indicate statistically significant differences between days of preservation, while lowercase letters denote statistically significant differences between treatments on each sampling day, with a significance level of *p* < 0.05.

Figure 2 illustrates the progression of TVB-N levels during storage. Concentrations increased across all treatments, most notably from day 3 onward. No statistically significant differences were observed between groups at either the commencement or conclusion of the storage period (23.73 ± 0.66 and 55.55 ± 13.98 mg TVB-N/100 g of sample, respectively). Although the CIBTS treatment exhibited a significant decrease (*p* < 0.05) on day 6, standard deviations within 8 mg% of non-protein nitrogen suggest that the parameter remained stable from day 6 to 12. This trend is further substantiated by its correlation with observed microbial growth patterns.

### 3.3. Melanosis Evaluation

**Figure 3:**
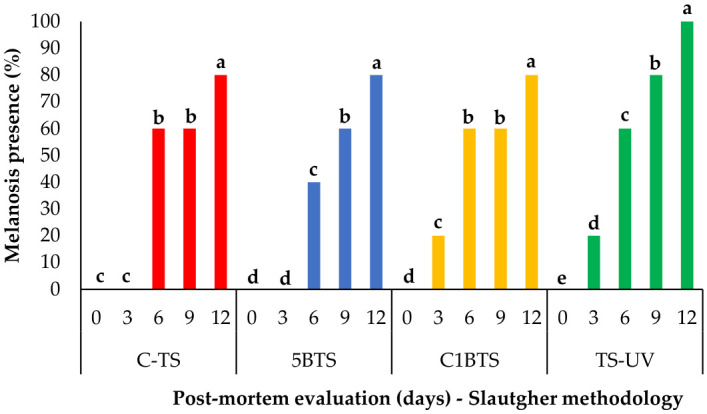
Melanosis (%) in refrigerated raw shrimp evaluated at day 0, 3, 6, 9, and 12 post-mortem using different slaughter methodologies (C-TS, thermal shock; 5BTS, 5 min bleach and thermal shock; C1BTS, clarifying, 1 min bleach, and thermal shock; and TS-UV, thermal shock and UV treatment). Lowercase letters represent significant differences inside each group (*p* < 0.05).

A strong positive correlation was observed between melanosis scores assessed in raw and cooked shrimp, with a Pearson correlation coefficient of r = 0.988, indicating an almost perfect agreement between both evaluations. This result suggests that melanosis development was consistently captured regardless of the product state. Therefore, to avoid redundancy, only the results corresponding to raw shrimp melanosis are presented in the following sections. The progression of melanosis in shrimp subjected to different slaughter methodologies over a 12-day post-mortem storage period revealed notable differences over time and between treatments. As illustrated in Figure 3, all treatments exhibited a significant and progressive increase in melanosis levels from day 0 to day 12.

In shrimp subjected to C-TS, melanosis was absent during the first three days, followed by a sharp increase to 60% on day 6, reaching 80% by day 12. A similar pattern was observed in shrimp from the 5BTS treatment, but with a moderate increase to 40% on day 6. In contrast, shrimp slaughtered using C1BTS displayed an earlier onset of melanosis, already visible on day 3 (20%), followed by a sharp increase to 60% at days 6 and 9, and finally reaching 80% by day 12, matching the final values of C-TS and 5BTS. The most distinctive pattern was observed in the TS-UV group, and although no melanosis was observed on day 0, signs of melanosis appeared on day 3 (20%), followed by an increase to 60% at day 6, an increase to 80% at day 9, and peaking at day 12, representing the highest final melanosis level across all treatments.

**Figure 4:**
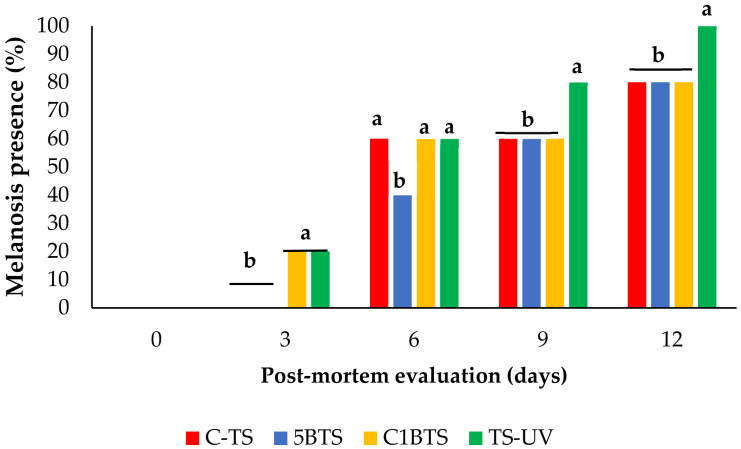
Presence of melanosis in shrimp evaluated at day 0, 3, 6, 9, and 12 post-mortem in refrigerated storage, slaughtered using different methodologies (C-TS, thermal shock; 5BTS, 5 min bleach and thermal shock; C1BTS, clarifying, 1 min bleach, and thermal shock; and TS-UV, thermal shock and UV treatment). Lowercase letters represent significant differences between groups (*p* < 0.05).

Comparative analysis between treatments at each time point (Figure 4) further highlights the impact of slaughter methodology on melanosis development. On day 3, both C1BTS and TS-UV groups showed initial melanosis signs (20%), while 5BTS and C-TS remained at 0%. From day 6 onward, melanosis levels across treatments were more similar, although 5BTS remained slightly lower. On day 6, melanosis levels reached 60% in all treatments except for 5BTS, which reached this threshold on day 9. The 60% score represents a critical point of rejection by the panel of trained judges. By day 12, all treatments reached high melanosis values (80–100%), with TS-UV showing the most severe progression.

### 3.4. PCA Analysis

**Figure 5:**
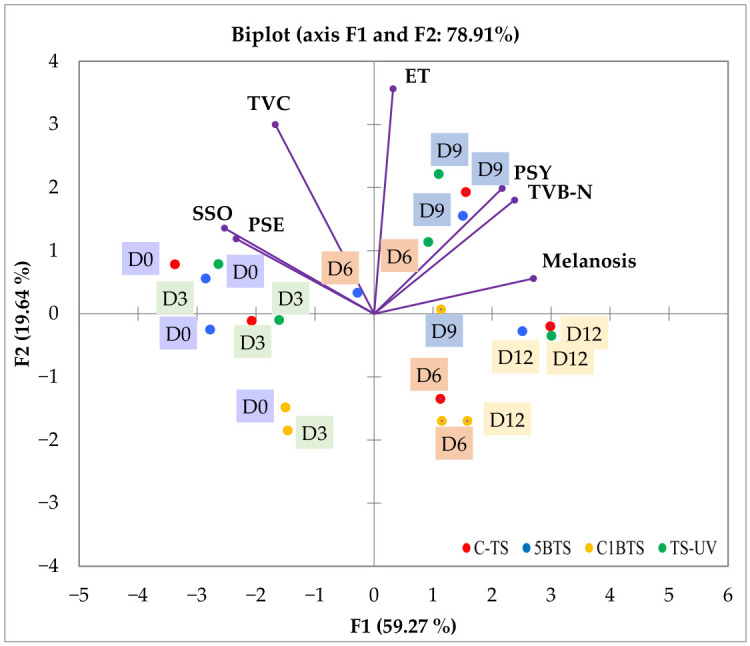
PCA analysis for the microbiological, physicochemical, and melanosis evaluations for each of the slaughtering methods tested (C-TS, thermal shock; 5BTS, 5 min bleach and thermal shock; C1BTS, clarifying, 1 min bleach, and thermal shock; and TS-UV, thermal shock and UV treatment) on the different post-mortem days (D0, D3, D6, D9, and D12) in refrigerated storage.

The PCA provided an integrated view of how microbiological growth, physicochemical alteration, and melanosis evolution jointly characterized the post-mortem quality trajectory of *P. vannamei* during refrigerated storage (Figure 5). The first two components explained nearly 80% of the total variance, with F1 (59.27%) acting as the main axis discriminating against the different deterioration stages, confirming that the selected variables captured the overall spoilage trajectory effectively.

Variables associated with advanced spoilage, particularly PSY counts and TVB-N, clustered on the right side of F1 and showed clear co-location with day 9 of storage. These variables also aligned with melanosis scores and day 12, indicating that all three reached their highest levels during the final stage of storage. In contrast, SSO and PSE were positioned in the opposite quadrant and were primarily associated with days 0 and 3 post-mortem, reflecting their higher prevalence in the early stages of quality decline. Meanwhile, ET and TVC were clustered near the origin of the PCA, suggesting weak discriminatory power across sampling times or deterioration stages.

### 3.5. QDA Analysis

**Figure 6:**
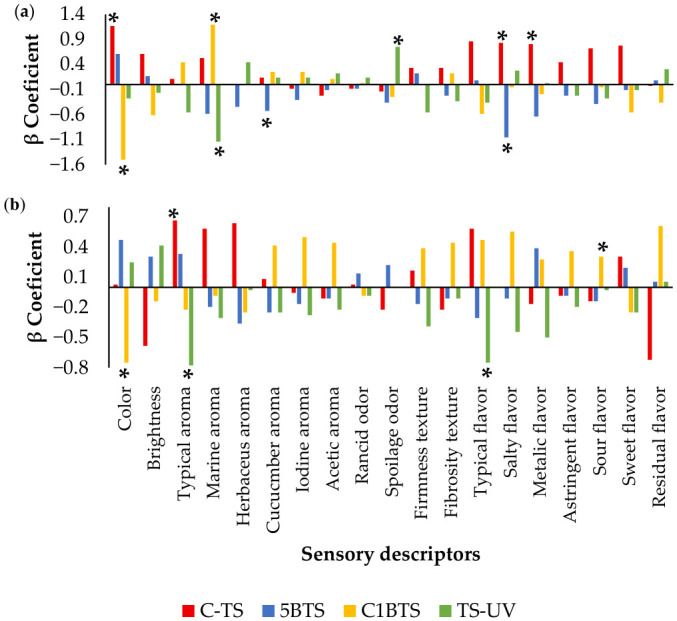
β coefficients obtained for the sensory descriptors evaluated in shrimp slathered by different methodologies (C-TS, thermal shock; 5BTS, 5 min bleach and thermal shock; C1BTS, clarifying, 1 min bleach and thermal shock; and TS-UV, thermal shock and UV treatment) evaluated at (**a**) one and (**b**) seven days, based on the intra-treatment analysis. Asterisk (*) symbol represents significant difference between sensory descriptors in each treatment (*p* < 0.05).

The QDA analysis provided valuable insights into the shrimp’s sensory profile and a detailed characterization of the cooked product at one and seven days post-mortem. An intra-treatment evaluation enabled the panel to identify key sensory descriptors that significantly differentiated the shrimp samples according to the killing method applied (Figure 6a and Figure 6b for days one and seven, respectively). Based on the analysis, shrimp from the C-TS group were characterized on day one by more intense color, and prominent salty and metallic flavors; and by day seven, the typical aroma became their most distinguishing feature. In contrast, the 5BTS treatment exhibited a clear absence of cucumber aroma and salty flavor at day one and maintained a generally flattened sensory profile by day seven, with no distinctive attributes. Shrimp from the C1BTS group showed a pronounced marine aroma on day one and a marked sour flavor on day seven but lacked noticeable coloration in both assessments. Lastly, the TS-UV treatment was characterized by the absence of marine aroma and the presence of spoilage odor on day one, followed by a reduction in both typical aroma and flavor at day seven, resulting in a negative overall evaluation.

**Figure 7:**
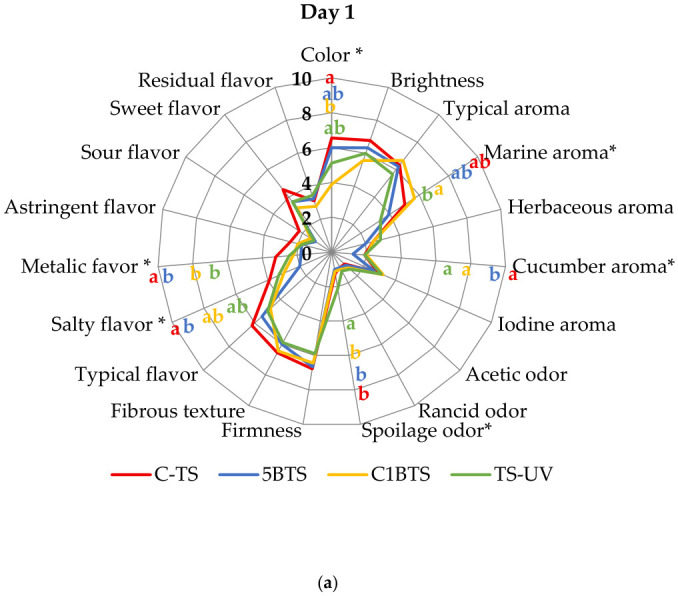

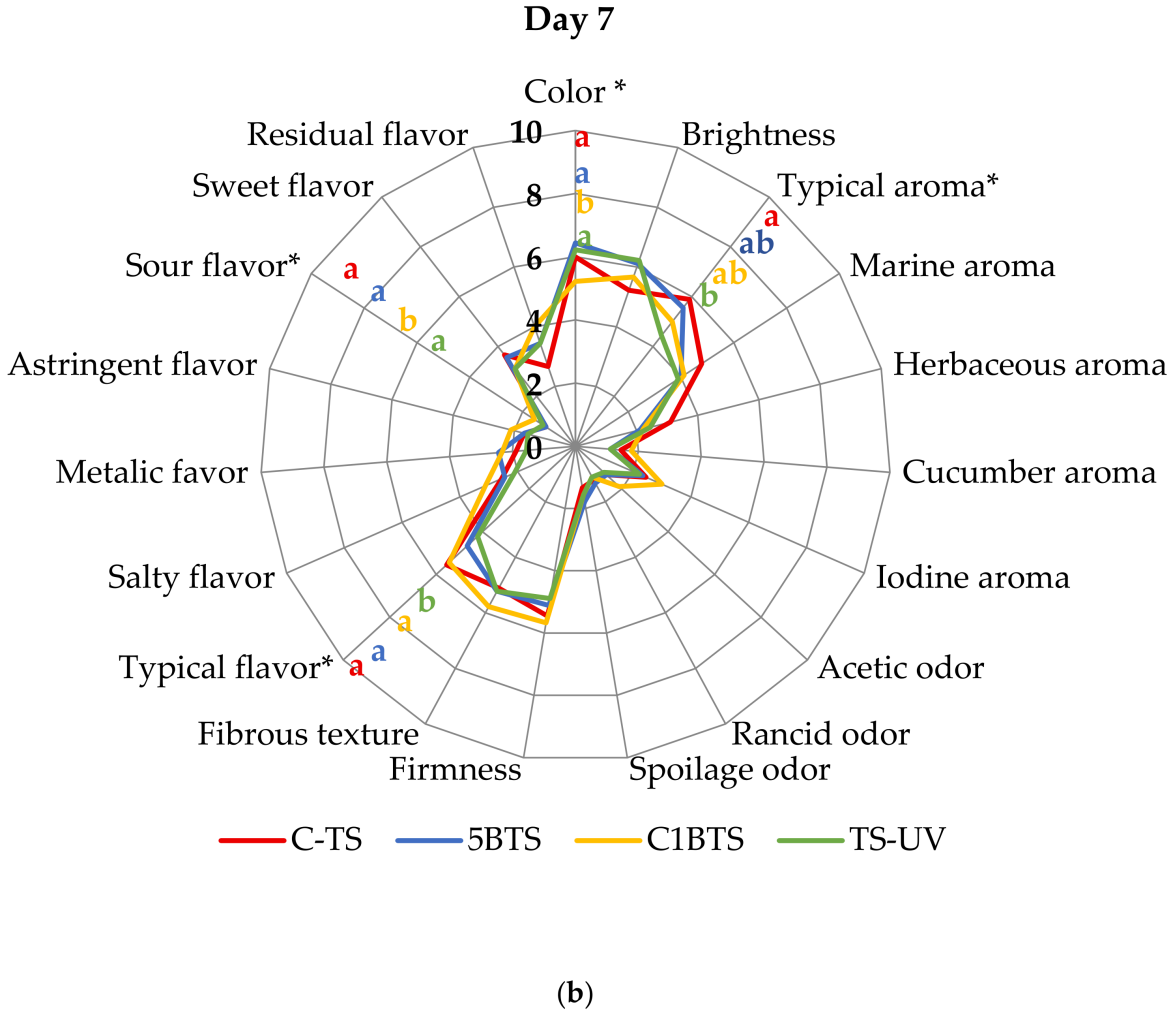
Shrimp sensory profile slathered by different methodologies (C-TS, thermal shock; 5BTS, 5 min bleach and thermal shock; C1BTS, clarifying, 1 min bleach, and thermal shock; and TS-UV, thermal shock and UV treatment) evaluated at (**a**) one and (**b**) seven days post-mortem, based on the intertreatment analysis. Asterisk (*) symbol represents significant difference between treatments (*p* < 0.05). Lowercase letters denote statistically significant differences between treatments with a significance level of *p* < 0.05.

Figure 7a,b illustrate the sensory profiles of shrimp evaluated at one and seven days post-mortem intertreatment, respectively. In general, all treatments exhibited comparable sensory characteristics at both times. High scores were consistently attributed to desirable attributes, such as color, brightness, typical aroma, firmness, fibrous texture, and the characteristic flavor of the meat. In contrast, descriptors such as herbaceous, cucumber, and iodine aromas, as well as acetic, rancid, and spoilage odors; metallic flavor; astringency; and sourness, received low ratings across treatments.

Focusing on day 1 post-mortem (Figure 7a), shrimp from the C-TS group showed the highest score for color, significantly surpassing C1BTS, and also stood out in regard to salty and metallic flavor, outperforming 5BTS. For marine aroma, C1BTS recorded the highest score, significantly higher than TS-UV and comparable to both C-TS and 5BTS. Regarding cucumber aroma, shrimp from the 5BTS treatment exhibited the lowest score. At day 7 post-mortem (Figure 7b), color remained a relevant attribute, with C1BTS again scoring the lowest. Significant differences emerged in typical aroma, with C-TS registering the highest value, significantly superior to TS-UV, though not different from 5BTS and C1BTS. Typical flavor also showed significant differences, with TS-UV obtaining the lowest score. Additionally, sour flavor became significant, with C1BTS receiving the lowest rating for this attribute.

## 4. Discussion

This study demonstrates that the post-mortem quality evolution of *Penaeus vannamei* is shaped by a complex interaction between the microbial background of BFT systems, slaughter methodology, and refrigerated storage. Microbial activity is the primary driver of seafood spoilage [57], and the elevated initial counts observed in the present study, regardless of the treatment, reflect the intrinsically higher microbial load of BFT compared with clear-water systems [58]. However, BFT provides recognized production advantages, including improved growth performance, feed efficiency, and water quality [59]. This was reflected in TVC counts, whose initial values exceeded those commonly reported for *Sparus aurata* (3 log CFU/g; [60]) and *Litopenaeus stylirostris* (3.5 log CFU/g; [61] and 4 log CFU/g; [62]). Therefore, slaughter methodology modulated early microbial dynamics but did not markedly alter TVC trajectories, indicating that the cultivation system defines baseline microbial, while post-harvest handling governs subsequent evolution. PSE exhibited partial agreement with classical spoilage dynamics, remaining stable during the first three days, followed by decline, as reported in *Sparus aurata* [63], but contrasting with previous studies of *P. vannamei* and *Penaus notialis* [64,65,66]. In contrast, ET followed expected patterns for fresh shrimp [64], with similar concentrations and a limited response to slaughter methodology beyond the initial storage phase. Although C1BTS reduced ET counts by approximately 1 log CFU/g immediately after slaughter, likely due to intestinal clarification combined with sodium hypochlorite exposure, this initial reduction was transient and disappeared after three days of storage. This suggests transient impact, as described in bivalve mollusks, where a reduction in their microbial load occurs when subjected to a depuration process [67].

Unexpectedly, SSOs decreased over storage, deviating from the classical spoilage *Shewanella*-dominated model [68]. This likely reflects ecological competition within BFT systems rather than an absence of spoilage activity. A similar trend was observed in the ET treatment between days 9 and 12 post-mortem, where bacterial concentrations decreased following an increase. Biofloc microbial communities are known to modulate microbial dynamics through mechanisms such as nutrient competition, quorum sensing interference, and bacteriocin production [69]. These interactions may reduce the ecological fitness of dominant spoilage organisms, such as *Shewanella* or *Enterobacter* spp., within biofilms, potentially redistributing spoilage activity among other microbial taxa.

In relation to PSY, it emerged as the key group driving spoilage progression. Initial counts were consistent with the literature, yet growth was moderate, reaching substantially lower concentrations than previous reports [46,70]. Importantly, PSY development was clearly modulated by slaughter methodology. TS-UV shrimp consistently exhibited higher PSY, suggesting insufficient antimicrobial efficacy or cold chain disruption due to extra handling. In contrast, the C1BTS treatment effectively limited late PSY proliferation, indicating that intestinal clarification prior to thermal shock can reduce the microbial load. These results highlight that antimicrobial interventions interact with handling and pre-mortem stress, generating treatment-specific responses.

Considering the abovementioned information, the microbial analysis evidenced the shelf life of untreated fresh *P. vannamei* up to 12 days, since none of the slaughter methodologies exceeding the 7 log CFU/g threshold considered acceptable for fresh products intended for human consumption (ICMSF, 1986) [71].

Consistent with microbial activity, TVB-N increased progressively in all treatments, reflecting post-mortem protein degradation and amine accumulation [72]. Initial values (~25 mg/100 g) were higher than those typically reported for untreated *P. vannamei* [65,73,74]. Although slaughter methodology influenced early TVB-N progression, particularly in TS-UV, by day six, all treatments exceeded the European freshness limit for fish (30–35 mg N/100 g; Commission Regulation (EC) No. 1022/2008) [50]. However, currently, there is no specific TVB-N limit for shrimp in the EU regulation. At the end of the trial, concentrations converged across treatments ~55 mg/100 g, being comparable to previous studies [65,73,74]. This confirms that, under additive-free conditions, fresh refrigerated *P. vannamei* should ideally be consumed within six days post-harvest, despite microbiological acceptability extending beyond this period. As such shelf lives are often impractical in commercial distribution, complementary preservation strategies, such as chitosan-based edible coatings or biopreservative formulations, remain essential for extending freshness without compromising product integrity [65,74].

Regarding melanosis development, it increased progressively in all treatments, consistent with prophenoloxidase activation response of shrimp tissues under chilled storage in the absence of chemical inhibitors [75,76]. As described in the literature, several *pre-mortem* biological factors, including capture method, molting stage, and harvest season, modulate melanosis development [77,78]. Slaughter methodology clearly shaped the onset. TS-UV and C1BTS exhibited black spots after three days of storage. TS-UV produced the fastest and most intense melanosis, reaching 100% by day 12, likely due to the UV-C-induced oxidative stress via free radical formation that is widely documented in muscle foods [79,80]. And the earlier onset in C1BTS likely reflects prolonged handling required for intestinal clarification, indicating that extensive pre-mortem handling promotes faster activation of melanosis pathways [81].

In contrast, 5BTS delayed melanosis onset, possibly via transient melanosis-related enzymatic inhibition. Although both C1BTS and 5BTS involved hypochlorite exposure, their effects differed markedly: the minimal handling in 5BTS contrasts with the extended pre-slaughter period in C1BTS, likely negating any enzymatic inhibition by hypochlorite. These observations contrast with previous findings [82], where black-spot developed after 3 days of chilled storage in fresh *P. vannamei* treated with 40 ppm sodium hypochlorite for 10 min. The use of a slightly higher concentration (50 ppm) and shorter exposure time in 5BTS, combined with minimal manipulation, may explain the further delay observed here. Considering a 60% melanosis score as the rejection threshold [16,83], 5BTS provided approximately three additional days of acceptable appearance compared with the other treatments.

The PCA provided an integrated perspective linking microbiological, chemical, and melanosis quality indicators. The close clustering of PSY, TVB-N, and melanosis, within days 9 and 12, confirmed their shared deterioration pathway. This agrees with the well-established spoilage sequence in chilled shrimp, where PSY proliferation promotes proteolysis and deamination, leading to TVB-N accumulation [84,85]. Previous research has reported strong positive associations between PSY and TVB-N, supporting PSY as a sensitive indicator of mid-to-late-stage quality loss [84,86]. The co-occurrence of elevated PSY and TVB-N, and melanosis at later storage stages further suggests that black-spot development is intertwined with microbial and chemical spoilage processes, as indirectly demonstrated by antioxidant and antimelanotic treatments simultaneously attenuating TVB-N and melanosis progression [62,87,88]. Collectively, these findings reinforce the notion that melanosis should be viewed as an integral component of a broader post-mortem deterioration process driven by microbial and biochemical activity.

The inverse relationship between specific SSOs and TVB-N observed likely reflects ecological interactions inherent to BFT systems. Quorum sensing and competition may suppress classical SSOs, including *Shewanella* spp. and *Aeromonas* spp., despite the ongoing biochemical spoilage [89,90,91]. Within biofloc-based shrimp farming, taxon-specific evidence from the literature helps to explain the strong coupling between PSY and TVB-N alongside the apparent decoupling of SSOs. *Vibrio* spp. and *Pseudoalteromonas* spp. are consistently reported as dominant [92], many of which tolerate chilling conditions and contribute substantially to the psychrotrophic fraction most closely linked to TVB-N accumulation. Competitive and facilitative interactions among these taxa have been documented, including anti-*Vibrio* activity by *Pseudoalteromonas* [93], and synergism between *Pseudoalteromonas* spp. and *Psychrobacter* spp. that enhances spoilage, as previously described in smoked salmon [94]. Such dynamics may redistribute spoilage potential across the microbial community, providing a plausible explanation for the observed increase in TVB-N concurrent with elevated psychrotrophic activity, despite limited growth of traditional SSOs. ET and TVC, which were positioned near the PCA center, exhibited weak associations with storage stages or quality parameters, indicating that their presence did not drive the principal quality changes. This aligns with previous findings indicating that ET and TVC are unreliable predictors of sensory deterioration in shrimp [64].

From a practical perspective, PCA highlights that spoilage in biofloc-reared shrimp is primarily driven by microbial community shifts rather than dominance of a single classical group. The strong coupling of PSY, TVB-N, and melanosis suggests that monitoring PSY alongside chemical freshness indices may provide a better early warning of quality loss than SSO enumeration alone. The integration of non-destructive TVB-N estimation [95], microbiome management [96], and antimelanotic strategies such as modified atmosphere packaging and antioxidant coatings [97] could strengthen control.

Additionally, when all slaughter methods were considered, the PCA showed no distinct clustering among treatments, indicating comparable deterioration trajectories. Storage time was the dominant factor governing quality decline, shifting from early freshness (days 0–3; SSOs and *Pseudomonas* spp.) to advanced spoilage stages (days 9–12; PSY, TVB-N, and melanosis). Although slaughter method influenced specific parameters, particularly melanosis onset, it did not alter the general spoilage pathway, which was mainly governed by time–temperature interactions. Therefore, this multivariate approach underscores the importance of interpreting melanosis within the broader context of post-mortem quality decline, especially when slaughter practices affect appearance without major shifts in microbial ecology.

The QDA analysis provided critical insight into the sensory performance and potential limitations of fresh shrimp handled under different slaughter methodologies. The overall homogeneity of the sensory profiles indicates high quality of the fresh, untreated, refrigerated product and suggests that none of the methods severely compromised the sensory attributes. This supports the sensory quality of biofloc-reared shrimp, comparable to that reported for seawater systems [98], despite the absence of clear-water control. Nevertheless, the sensory analysis revealed differentiated effects of slaughter methodology on specific attributes, both immediately after processing (day 1) and following refrigerated storage (day 7).

On day 1, the sensory profile was defined by color, marine and cucumber aroma, spoilage odor, and metallic and salty flavors. The pre-mortem clarification negatively affected shrimp coloration in the C1BTS treatment, which scored lower than C-TS treatment. This result contrasts with earlier findings [99], where no relevant differences in reddish coloration were observed after intestinal sludge cleaning, suggesting that the discoloration observed here was likely attributable to handling-related factors and residual chlorine. Free chlorine residues have been linked to discoloration and off-flavors in seafood [100,101]. However, residual chlorine levels in the present study were consistently below 1 ppm after treatment due to the rinsing effect of the ice slurry, indicating that any observed sensory differences are more likely related to short-term surface interactions or handling conditions rather than residual chlorinated compounds. A similar pattern was observed for cucumber aroma, with the lowest scores in 5BTS, indicating that inadequate control of residual chlorinated compounds may dull sensory attributes [101,102]. These findings suggest the need for additional rinsing or more frequent renewal of the ice slurry when chlorine treatments are used. With respect to marine aroma, shrimp subjected to UV radiation exhibited significantly lower scores than the other treatments, and higher spoilage odor, indicating that the UV-C treatment was insufficient, consistent with reports of reduced aroma intensity depending on dose and matrix composition [103,104]. This limited effectiveness may be explained by the relatively low and estimated irradiance applied, the short exposure time, and the inherent constraints of UV-C penetration in complex food matrices such as shrimp, where surface shielding and irregular geometry can significantly reduce antimicrobial efficacy. In relation to metallic and salty flavor, the C-TS treatment exhibited significantly higher scores. The metallic notes are associated with early lipid oxidation and volatile aldehydes and ketones [105,106,107]. These results suggest that thermal shock alone may induce oxidative pathways through physical stress mechanisms. Additionally, the salty flavor is likely attributable to variations in ionic exchange dynamics during handling in iced water, consistent with evidence that modest changes in NaCl concentration can substantially modulate perceived saltiness and water retention in seafood products [108,109].

By day 7, clearer differentiation among slaughter methods emerged, with color, typical aroma, sour flavor, and typical flavor as the most discriminant attributes. Color remained significantly lower for the C1BTS, reinforcing the need for stricter control of chlorine control. Typical aroma was highest in C-TS and lowest in TS-UV, with intermediate values for C1BTS and 5BTS, suggesting that disinfectant or photochemical hurdles may attenuate desirable odor-active compounds. The fact that chlorine-treated samples did not reach the same typical aroma scores as the C-TS group suggests that chlorine-mediated surface reactions may be perceptible during retronasal evaluation (tasting) while remaining less evident in orthonasal perception (headspace odor). This divergence has been documented when residual chlorination levels approach sensory-detection thresholds [101]. Typical flavor followed a similar pattern, with the lowest values in TS-UV, consistent with previous UV studies [103,104]. Finally, sour flavor was lowest in the C1BTS, whereas no differences were observed among the remaining methods. This pattern aligns with the expectation that clarification in chilled seawater combined with low-ppm NaClO constitutes the most effective protocol in suppressing acidogenic psychrotrophs and delaying organic acid accumulation [102,110].

Taken together, the QDA results indicate that slaughter methodology modulates specific sensory attributes of cooked shrimp without necessarily compromising overall product quality. The C-TS treatment preserved a sensory profile closely associated with freshness, likely due to rapid death with reduced stress, resulting in moderate microbial control and retention of positive attributes. Among hypochlorite treatments, 5BTS produced a neutral profile, possibly due to the 5 min disinfectant exposure reducing surface volatiles without overt defects; this approach delayed melanosis and extended shelf life to about 9 days. C1BTS achieved stronger microbial control but showed earlier melanosis and intermediate sensory stability, suggesting that prolonged handling increased pre-mortem stress and accelerated post-mortem changes. TS-UV performed worst, with higher PSE and PSY, early melanosis, and the lowest sensory scores, indicating insufficient microbial control and probable oxidative damage. These findings highlight the need for multidimensional evaluation of slaughter practices and provide a framework for optimizing post-harvest handling in biofloc-based shrimp systems.

## 5. Conclusions

This study demonstrates that slaughter methodology is a critical determinant of post-mortem quality in fresh, additive-free *Penaeus vannamei* reared in biofloc systems, where spoilage dynamics are strongly shaped by the distinctive microbial background of BFT and the activity of psychrotrophic bacteria. While storage time and temperature define the general deterioration pathway, the choice of slaughter method modulates the onset and intensity of key quality attributes, particularly melanosis development, sensory acceptance, and microbial proliferation. Among the evaluated strategies, the 5BTS method (5 min sodium hypochlorite, followed by thermal shock) emerged as the most effective approach, providing a balanced control of quality loss by delaying melanosis, maintaining acceptable sensory properties, and preventing critical microbial growth throughout refrigerated storage. This performance highlights 5BTS as a robust and practical option for preserving the commercial value of minimally processed shrimp without the use of chemical preservatives.

## Data Availability

The original data presented in the study are openly available in institutional repository RiuNet (Universitat Politècnica de València) at https://riunet.upv.es/handle/10251/235077.

## Supplementary Materials

The following supporting information can be downloaded at https://www.mdpi.com/article/10.3390/foods15101695/s1. The supplementary material consist of the sensory quality evaluation score sheet for farmed shrimp.

## Abbreviations

The following abbreviations are used in this manuscript:

BFT	Biofloc technology
C-TS	Control—thermal shock
5BTS	Bleach + thermal shock treatment
C1BTS	Clarification + bleach + thermal
TS-UV	Thermal shock + UV
TVB-N	Total volatile nitrogen compounds
UPV	Universitat Politècnica de València
UNIZAR	University of Zaragoza
QDA	Quantitative Descriptive Analysis
ISO	International Organization for Standardization
TVC	Total viable mesophilic bacteria
PSY	Psychrotrophic bacteria
ET	*Enterobacteriaceae*
SSO	Standard spoilage organism
PSE	*Pseudomonas* spp.
Log CFU/g	Colony-forming units per gram
PCA	Principal Component Analysis
