# Supplementary material for: Effects of Slaughter Methods on the Quality and Refrigerated Shelf Life of Biofloc-Cultured White Shrimp (Penaeus vannamei)
Source: Foods. 2026 May 12;15(10):1695. doi: 10.3390/foods15101695 (PMC13205315; doi:10.3390/foods15101695)
Supplement: Supplementary file 1 [file foods-15-01695-s001.zip › foods-4296104-supplementary.pdf]

GAB Grupo de Acuicultura y Biodiversidad

Instituto Universitario de Investigación Mixto Agroalimentario de Aragón
Universidad Zaragoza

SENSORY QUALITY EVALUATION OF FARMED SHRIMP

With the shrimp laying down on a plate, analyze the following aspects:

Blackspot defect (melanosis progression)

0 2 4 6 8 10

Red-orange color intensity (aspect)

Faint Intense

Brightness intensity in the shell

Faint Intense

Shrimp evaluation (AROMA)

Characteristic steamed shrimp aroma

Absent Intense

Marine aroma

Absent Intense

Freshly cut grass aroma

Absent Intense

Cucumber aroma

Absent Intense

Iodine odor

Absent Intense

Acetic odor

Absent Intense

Rancid odor

Absent Intense

Spoilage odor

Absent Intense

Once the whole cooked shrimp has been evaluated, the shell is removed for meat evaluation.

Shrimp evaluation at the mouth (TEXTURE)

Firmness (first bite with incisors)

Soft Firm

Fibrosity (in mouth, during mastication)

Nothing Much

Shrimp evaluation at the mouth (FLAVOR)

Remember to take deep breaths prior to each evaluation.

Characteristic flavor to shrimp (tasting and breathing with the sample)

Absent Intense

Wild flavor (salty-iodized)

Absent Intense

Metallic flavor

Absent Intense

Sweet flavor

Absent Intense

Astringent flavor

Absent Intense

Sour flavor

Absent Intense

Residual flavor (Immediately after swallowing the sample)

Absent Intense
